# Bibliographical

**Published:** 1858-09

**Authors:** 


### BIBLIOGRAPHICAL.

We have received from the author, and have had lying upon our table for a considerable length of time, the "*Institutes of Medicine*," by Martyn Paine, A. M., M. D., L. L. D., Professor of the Institutes of Medicine and Materia Medica in the University of the city of New York, &c. And also by the same author—

*The "Medical and Physiological Commentaries*;" and in addition, from the same source—

*Essays on the Philosophy of Vitality*," as contradistinguished from Chemical and Mechanical Philosophy, and the "modus operandi of Remedial Agents."

In these works are embodied the views of one of the most laborious and learned Medical Philosophers of this or any other age, in this or any other country, upon the complicated theories of Physiology, Pathology, and Therapeutics, in reference to the great principles and laws of organic being, in accordance with the doctrine, that to be enabled to erect a substantial fabric of Institutes which shall guide the hand of art, we must ascend progressively along the fundamental facts in these various departments, till at last we proceed to convert the great system to practical uses in the preservation of health, and a just, intelligible, and philosophical application of the Materia Medica to morbid states of the body.

Notwithstanding, however, our great respect for the author of these works, we do not desire to be understood as committing ourselves to his views, being as he is, the *peculiar* defender in this country, of what we conceive to be (as we understand them,) the erroneous doctrines of Solidism and Vitalism. But in the language of a respected confrere, "truth is not often found at either extreme of the arc of science, but loves rather to become the keystone which binds its centre; and so with these researches and discussions in reference to the fundamental principles of life; the man of experience—he who studies day by day the phenomena of health and disease—he who observes the results of practice, whether he claims to follow the ancient school of Galen, and the Humoralists, or the Hypocratic doctrines of Stahl, and the Solidists, will never be wise, if he forgets the hackneyed, but valuable axiom in "*medio tutissimus ibis.*"

We have neither the time or the space to attempt anything like a full or elaborate review of these works, but must conclude our very imperfect notice of them—after commending their contents in the most decided manner, as in the highest degree worthy of the most thorough investigation—by an extract from the appendix of a new edition of the "Institutes," recently published by Harper & Bro., of New York, in reference to the claims of certain gentlemen, (Drs. Allen of Michigan, and Campbell of Georgia,) who have recently figured largely as discoverers in Physiologial science, upon (as Dr. Paine intimates,) views and doctrines *long since* promulgated and put upon record by himself.

"That the Author's physiological and medical writings were generally known in Europe many years before the period at which "Dr. Campbell bases his claim" (1850,) is evident from the distinguished honors to which they had led in that Country before that period—that from the Medical Society of Prussia as early as 1842—that from the Medical Society of Leipsic in 1843; and the "Medical and Physiological Commentaries" of 1840 were published simultaneously in London and New York; and as to the United States, the *Commentaries* were early distributed throughout the land, and his *Institutes of Medicine* more than a year, also, before Dr. Allen's Lectures were delivered; and the Author's Lectures at the University, which form the groundwork of his Institutes, had been listened to annually by Medical Students from all quarters of the Union since the year 1841. In 1848 the Author applied the doctrine of reflex nervous action to a physiological demonstration of the substantive existence of the Soul and Instinctive Principle, which was then published in pamphlet form, and in 1840 the work was extended and assumed the shape of a book, and is now incorporated, in its essential parts, in these Institutes.

"Nor is that all; for the whole of this doctrine of reflex nervous action, and of the operation of the nervous power as an *alterative* and *excitant of the secretions* and of vascular action (both direct and reflex,) a *depressent* and *sedative* (according to the nature of exciting causes), and the *great immediate cause of diseases and their cure*—variously modifying organic actions—was set forth extensively and circumstantially in an "*Essay on the* MODUS OPERANDI OF REMEDIES" in 1843, of which the Author distributed, at that time, a large number of copies in London, and addressed four thousand copies to Physicians throughout the United States. The Author not only sent a copy of the work to Dr. Hall, but dedicated it to him (along with Prof. J. Müller and Dr. A. P. W. Philip) in connection with an "*Essay on the Philosophy of Vitality*;" and he may add that he controverted, in the former Essay, doctrines of Dr. Hall (in "*Memoir on Diseases and Derangements of the Nervous System*, 1841"), which were in direct opposition to those which are now in question (also, p. 296—297, § 476½, *b*). These Essays were subsequently bound up in the Third volume of the "*Medical and Physiological Commentaries*," where the former may be readily consulted. But Dr. Philip had fully deduced from his experiments the *sedative* as well as *exciting* influence of the nervous system upon vascular action before Dr. Hall's experiments were made (§ 492).

"As to M. Bernard, his experiments bearing upon the connection of the nerves with the function of secretion, however much they may have been varied and multiplied, were anticipated long before by those of A. P. W. Philip, which are quoted extensively in these Institutes (p, 290–221), and towards which Dr. Hall had no friendly disposition (p. 306–308 and where the writer has controverted his views). The merit of originality which belongs to the present writer, in relation to these experiments, consists in their extensive application in illustrating the functions of the nervous power as a vital agent, profoundly interested not only as an "excito-secretory" power, and as a modifying cause of all secreted products, &c., when diverted from their natural standard, but in deducing from them a universal agency of the reflex action of the nervous system, through "the double nervous arc," in the production and cure of disease, and which he laboured to explode the chemical and physical doctrines as early as 1840. But, that the writer may not be misapprehended, he will say that he endeavours to establish the fact that secretion in animals, as in plants, is conducted by powers implanted in every part, but that it is constantly influenced physiologically, pathologically, and therapeutically, by reflex action of the nervous system.

"The writer is very sensible that unaccountable coincidences often present themselves in the development of new thoughts, and in the discovery of hidden things, especially where enduring reputation may be won. "*Ubi mel ibi apes.*"—"*Uno tiene la fama, y otro carda la lana.*" But the reader, with these Institutes before him, will quickly find that much that is claimed by Dr. Hall, and all that he has granted to Dr. Campbell in the foregoing quotation, and, therefore, all that Dr. Allen appropriates to himself,* abounds in this volume and, in fact, constitutes the life and soul of the work as it does, also, of the *Commentaries*," and of the Essay on the "*Modus Ope-*

---

* "Unus utrique

Error; sed variis illudit partibus."—HORACE.

*randi of Remedies;"* nor can the reader fail of the conclusion that, were Dr. Hall's "adjudication, and Dr. Allen's after-thought, founded in any justice, and were not the claimants themselves the obnoxious parties, the present writer would have been long ago convicted by them and by others of arrogant assurance and the grossest plagiarisms. Nevertheless, the Author is most happy to find that his solitary position is becoming relieved, and that a practical direction has been given to his labours by others which cannot fail of carrying forward the great doctrines at which he has toiled, and against manifold obstacles, during his professional life."

"*Mind and Matter, or Physiological Inquiries.*" By Sir Benjamin Brodie, Bart. D. C. L., Vice President of the Royal Society, London, with additional notes by an American editor.

We have received the above work from Messrs. S. S. & W. Wood, 380, Broadway, New York, through the hands of Wm. Kay, Bookseller of Atlanta.

In this Book is comprised a series of Essays, intended to illustrate the mutual relations of the physical organization and the mental faculties.

"The subject, although replete with interest and of much practical information, is one, as to which we have no means of obtaining such complete and definite knowledge, as to admit of its being presented in the shape of a systematic treatise. Some points may be considered as established with a sufficient degree of certainty. There are others as to which opinions may reasonably differ, while there are still a greater number as to which we must be content to acknowledge that, with our limited capacities, we have no means of forming an opinion at all."

To those who are interested in such inquiries, we think this book will prove interesting and profitable—the name of the author alone is sufficient guarantee, that it is at least a sensible work.

We have been specially pleased with some of the views presented, in regard to that *greatest* (if possible,) *humbug of the age*, *Phrenology*, an extract in reference to which is appended. After some allusion to instances of shrewd observations on character, made by Phrenologists, it is remarked :

"But I might also refer to still more numerous instances in which the phrenological examination of the head has proved to be a failure. You may perhaps regard me as being in some degree a prejudiced witness, and I will therefore merely refer you to an account, published some years ago, of the visit of Dr. Gall, the inventor of the science, to Sir Francis Chantrey's studio; when he pronounced the head of Sir Walter Scott (who had not the smallest turn for mathematics to be that of a great mathematician; that of Traughton, the mathematical instrument maker,to be the head of a poet; and at the same time discovered the indications of a superior intellect in another head, the living proprietor of which had certainly as little claim as any man could possibly have to be thus distinguished.

But even if the errors of phrenology were less numerous than I believe them to be, that would not go far towards convincing me of the value of their art. It is not very difficult for a clever observer of human nature to form a notion of some part of a man's character in the course of a brief conversation with him; and an enthusiast in phrenology may very honestly persuade himself that he has obtained from the examination of his head that knowledge which he has really obtained from other sources. Then observe how comprehensive the faculties and propensities of the phrenological system are supposed to be. A large development of the organ of destructiveness in the head of Hare the murderer, explained how it was that he was led to murder sixteen human beings that he might sell their bodies.* But in the head of another person who never committed a murder, it is sufficient to find that it exists in combination with a disposition to satire, or to deface mile-stones; and in the beaver and squirrel, it explains how it is that these animals are impelled to cut and tear in pieces the bark, leaves, and branches of trees, for the innocent purpose of constructing their cabins and nests. So the large size of the organ of acquisitiveness not only leads one person to be a thief and another to hoard, but it also explains the habits of the spendthrift (who does not hoard at all); and it impels storks and swallows to return after their migrations to establish themselves, each succeeding year in the same locality. Following these examples, I do not see that a phrenologist can be much at a loss in finding a character for any individual suited to the peculiar configuration of his head. But observe further, if a difficulty were to occur, how easily it may be explained away by an ingenious phrenologist. If ever there was a race of thoroughly remorseless murderers in the world, such were the Thugs of India. Generation after generation they were born and bred to murder. They looked to murder as the source not only of profit but of honor. Dr. Spry sent the skulls of seven of these demons, who had been hanged at Saugor, to some phrenological friends in Scotland. To their suprise, destructiveness was not a predominant organ in any one of them. But the anomaly was soon explained. The Thugs, it was said, had no abstract love of murder, but murdered for the sake of robbery.† It would not be easy to show that there was any difference between the Thugs and Hare, or Burke, or Bishop, in this respect."

"*Nature and Art in the Cure of Disease.*"—We have received from Messrs. Samuel S. & William Wood, of New York, through the hands of Wm. Kay, bookseller, of this city, a work entitled "Nature and Art in the Cure of Disease," by Sir Jno. Forbes, M. D., &c., &c. from the Second London Edition. This is a work of great merit, upon

* A System of Phrenology, by George Combe, 5th edition. vol. i. 262, &c.
† India, Pictorial and Historical, London, 1854, p. 356.

a most important subject; and while we do not undertake to endorse every thing it contains, we are satisfied (however much it may be opposed to the views of the great army of *Druggers*,) that in it may be found much more of truth than error.

The *great and crying evil* connected with the Medical profession, in our humble judgment, is, the idea upon the part of the public, and to a large extent upon the part of practitioners of medicine, that the practice of a Physician consists in little else than the prescription and administration of drugs.

However humiliating and degrading this view of the subject may be, we intend to deal plainly and emphatically with whatever we conceive to be grave errors, either in the profession, or those affecting its welfare from without. It is unquestionably to the idea that disease or derangement of the body, must of necessity be met by the administration of drugs, that we are indebted for a large proportion of the quackery of almost innumerable kinds, that curse the land.

It is to the prevalence of the idea that "Disease is an entity destructible by the introduction into the system of an appropriate *remedy*," that we are largely indebted for the horde of ignorant and unprincipled practitioners of all sorts of medicine, who are infesting the land.

It is to the want of appreciation and acknowledgment upon the the part of a large number in the profession, and the mass of mankind in general, of the wonderful capacities of the human body for resistance to disease and self-recovery from even the most serious disturbances, that we are indebted for many "*skillful doctors*" and "*accoucheurs*," who are literally ignorant of the first principles by which the human body "lives and moves and has its being." It is to the idea, that disease is to be *killed outright* by the administration of some potent *drug—specifics* for "all the ills that flesh is heir to" being scattered throughout the world, if we can only *accidentally* discover them—that we are indebted for the innumerable list of "*cures*" for the thousand and one maladies of the human race; it is to this that we are indebted for the inquiries: "*Do you profess to be good on Fits?*" "Does this *cure* Whooping Cough?" or, "that *prevent* Scarlet Fever?" It is to this that we are indebted for the successful *imposture* constantly practiced upon the credulity of the world, by those who either ignorantly or wilfully appropriate to themselves, powers and capacities in resisting and removing disease with which the Creator has endowed each living body. We are happy to know, however, that the glorious light which Physiology is throwing upon the science of

Medicine, is gradually dispelling the clouds of mist and error which have so long hung around it. Witness the admirable conservative power of Nature, as exhibited in the most palpaple manner, in the "Excretion" of useless and offensive materials from the body—the striking capacity of one organ to assume the duties of another, whose function has become deranged. Verily, in the language of the eminent Draper, "The great Physicians of the future, are to be the great Physiologists."

In giving such decided expression to our views, it would seem, perhaps, necesssary, that we should say that, in our judgment, so far from depreciating the Medical Art, we intend to elevate it still higher; to raise it above the atmosphere of quackery and imposture—of *mere* pills, and potions, and powders, to an enlightened understanding of *the laws* which are concerned in the operations of the body, that in disease, we may be able, intelligently, to "modify the processes in which the malady consists, in such manner as to render them less dangerous to the integrity of the animal system, and more controllable by its inherent conservative, and reparative process." To place the Medical Art in its true position, it must be viewed in the light of an *aid* to the powers of Nature; in the language of our Author, "It must take up its true position, as a helper of man's infirmities; thus proving itself to be not simply useful, but most valuable in almost every case of disease, slight or severe, curable or incurable, its appliances being directed by a true knowledge of what Nature and Art respectively can and can not do, they can be made beneficially available in the most unpromising instances." We earnestly desire that this book could be scattered broadcast through the land, not only among the profession, but that it might fall into the hands of intelligent readers generally, believing, as we do, that it would largely contribute to the exposure and confusion of medical impostures of every description, and decidedly aid in the diffusion of a more just and accurate knowledge of the real nature of Disease, and the true character and power of the Medical Art.

"*A Manual of Psychological Medicine.*"—We have received from the Publishers, Messrs. Blanchard & Lea, Philadelphia, through the hands of Messrs. J. J. Richards & Co., Atlanta, a Manual of Psychological Medicine containing the history, nosology, description, statistics, diagnosis, pathology and treatment of *Insanity*, with an appendix of cases by Jno. Chas. Buckhill, M. D., and Dan'l. H. Tuke, M. D., of London.

In explanation of the origin of the work, the author makes the following statement in the preface :—"The authors of the following pages have long felt the want of a systematic treatise on Insanity adapted to the use of students and practitioners in Medicine.

Numerous monographs and works on limited portions of Psychological Medicine have appeared of late years. They are of great value to specialist Physicians, but they do not meet the oft repeated enquiry of the student and practitioner, "To what systematic treatise on Insanity can I refer ?"

Dr. Prichard's excellent "Treatise on Insanity" has undoubtedly been the one which hitherto has most nearly afforded the desired information; but it was written a quarter of a century ago, at a time when the treatment of Insanity bore an aspect entirely different to its present one; and moreover it is now out of print.

A knowledge of the nature and treatment of Insanity is now expected of every well educated medical man. The India Board require it of all persons to whom Medical appointments are given under their new system of competitive examination. It is reasonable to expect that the good example thus set will be followed in other quarters ; and a desire to obtain a competent knowledge of this important branch of Medical practice has become far more general in the profession than it ever before has been.

The authors are aware that no amount of reading can render it safe to dispense with a clinical knowledge of mental disease. Their aim has been to supply a text book which may serve as a guide in the acquisition of such knowledge, sufficiently elementary to be adapted to the wants of the student, and sufficiently modern in its views, and explicit in its teaching, to suffice the demands of the practitioner."

From the examination we have been able to give the work, it is our judgment that the authors have succeeded in supplying a *desideratum*, which, in reference to a medical book, is saying a great deal.

There is, we presume, no subject in the whole range of our comprehensive science, in reference to which, there is so much negligence manifested by the mass of the profession : this should not be, for while it is true there are few medical men who find themselves in favorable circumstances for the treatment of the insane, and as an almost universal rule it is best for the unfortunate subject of the malady, as well as the friends, that the sufferer should be placed in an Asylum, yet, no man can deserve the name of a scientific, and enlightened physician, who is not acquainted with, at least, the principles lying at the foundation of the treatment of this unfortunate class of our fellow men.

No man should be in the profession who is not a philanthropist ; and in the radical change which has taken place of late years in the management of the insane, is afforded one of the most gratifying triumphs of the benevolence and intelligence which so eminently distinguishes our noble science; and on this account the acheivements [illegible]ere detailed should commend themselves in the most decided manner, [illegible]o every true physician. From the insane having been treated as wild [illegible]easts, and consigned to a hopeless lunacy, it is now asserted upon high authority, "That with a well constituted governing body, animated by philanthrophy, directed by intelligence, and acting by means of proper officers (intrusted with a due degree of authority over attendants, properly selected and capable of exercising an official superintendance over the patients) there is no asylum in the world in which all mechanical restraints may not be abolished, not only with perfect safety, but with incalculable advantage." In conclusion we think we can recommend this work as full of interest and profit, not only to the physician, but to the philanthropist whether in or out of the profession.

It is a volume of 530 pages and includes the history, nosology, description, statistics, diagnosis, pathology and treatment of *Insanity*.

